# Assessing Physicians’ Adherence to the 2023 Sudan Malaria Case Management Protocol

**DOI:** 10.7759/cureus.89961

**Published:** 2025-08-13

**Authors:** Abubakr Muhammed, Abdulrahman Abbas Yusuf Mohammed, Mohammed Ali Mohammed Ali, Iyman Elsmani, Alaa Ibrahim Makarem Mohamed Elsetiha, Maaz Mohamed Abdelaal Salih, Hesham Hadeed, Alaa Elmutaz Mohamed Mahmoud, Muram Mustafa Mohamed Mohamed, Nahla Widatalla Abdalla Hamrawi, Athkar Yasir Hussien Abdalla, Abubakar Elkabashi, Mohaned Altijani Abdalgadir Hamdnaalla, Mohamed Elshafie, Hala Aamir Abdelraouf Ahmed, Mohammed Osman Ahmed Osman, Ola Abdoun Awad Abdulla, Fakher Aldeen Raft Fakher Aldeen Noman, Ayat Alfaki, Myada Omer Hussien Hamed

**Affiliations:** 1 Department of Surgery, University of Gezira, Madani, SDN; 2 Internal Medicine, Dongola Specialized Hospital, Dongola, SDN; 3 Internal Medicine, Hamad Medical Corporation, Doha, QAT; 4 Obstetrics and Gynaecology, Road El Farag General Hospital, Cairo, EGY; 5 Intensive Care Medicine, Dr. Sulaiman Al-Habib Medical Group, Riyadh, SAU; 6 Critical Care and Long-Term Care, NMC Royal Hospital, Abu Dhabi, ARE; 7 Cardiology, Lincoln County Hospital, Lincoln, GBR; 8 Accident and Emergency, RAK Hospital, Ras Al Khaimah, ARE; 9 Public Health, International University of Africa, Khartoum, SDN; 10 Internal Medicine, Sudan Medical Specialization Board, Khartoum, SDN; 11 General Practice, SouthDoc Services LTD, Cork, IRL; 12 Internal Medicine Specialist, Omdurman Islamic University, Khartoum, SDN; 13 Internal Medicine, The National Ribat University, Khartoum, SDN; 14 Internal Medicine, Andalusia Hospital, Jeddah, SAU

**Keywords:** anti-malarial agents, clinical audit, malaria, plasmodium falciparum, sudan

## Abstract

Background: Malaria represents a major public health challenge in Sudan. Adherence to national malaria diagnosis and treatment guidelines by healthcare providers is critical for effective disease control, preventing drug resistance, and ensuring optimal patient outcomes. This audit aimed to assess and improve physicians’ adherence to malaria guidelines.

Materials and methods: A two-cycle audit was conducted at Dongola Specialized Hospital, specifically targeting doctors' clinical practices. The first cycle (N=42) assessed baseline adherence among physicians to key standards, including protocol compliance, diagnostic accuracy, and appropriate treatment regimens for various Plasmodium species and patient groups (e.g., severe malaria, malaria in pregnancy). Following this, targeted interventions - such as physician training sessions and dissemination of updated clinical guidelines - were introduced. A second cycle (N=39) was then conducted to re-evaluate doctors' adherence and assess the impact of these interventions. Data were collected on predefined indicators and compared across both cycles.

Results: The first cycle identified the areas of low adherence, with general protocol adherence at 13 (30.95%), correct intramuscular (IM) artesunate injection site at 13 (30.95%), and second-line treatment for uncomplicated malaria in pregnancy (MIP) at 10 (23.81%). However, there was high adherence at 38 (90.48%) for the first-line treatment for P. falciparum. The second cycle demonstrated significant improvements across almost all standards. Protocol adherence surged to 35 (89.74%) (a 58.79% improvement), correct IM artesunate site to 31 (79.49%), and second-line treatment for uncomplicated MIP to 25 (64.10%) with 40.29% improvement. Intermittent preventive treatment of MIP with the correct drug sulfadoxine/pyrimethamine also saw a substantial increase to 36 (92.31%) with 49.45% improvement. Adherence to first-line artemether/lumefantrine for P. falciparum was maintained at 100%.

Conclusion: The audit findings confirm that the implemented interventions were effective in improving adherence to national malaria diagnosis and treatment guidelines in Sudan. The improvements across critical indicators highlight the importance of continuous training and auditing to improve the quality of malaria management and contribute to better patient outcomes and reduced disease burden in endemic settings.

## Introduction

Malaria remains a significant public health challenge, particularly in endemic regions like Africa. The Eastern Mediterranean Region (EMRO) witnessed a decrease in the incidence of malaria from 21 cases to 11 cases per 1000 population at risk [1]. This decrease is largely attributed to the adherence to preventive measures such as insecticide-treated bed nets, in-house spraying, and the availability of artemisinin-based combination therapy (ACT). In addition, the malaria vaccine represents a significant preventive advancement and has been rolled out in Sudan, further strengthening the impact of existing interventions [2].

Despite this, out of 22 countries and territories included in this WHO region, Sudan and Pakistan accounted for more than 80% of the cases [3]. In Sudan, the WHO Malaria Report 2024 revealed that malaria contributed to 0.16 deaths per 1000 of the population at risk, and the prevalence of artemisinin partial resistance has been confirmed [4]. Not only does malaria in pregnancy contribute to mortality, but it also plays a significant role in neurodevelopmental delays [5].

Effective malaria control requires robust diagnostic capabilities, reliable access to first-line antimalarial medications, and consistent adherence by healthcare providers to national treatment guidelines. However, these efforts are frequently undermined by the widespread practice of self-medication, which delays accurate diagnosis, promotes incomplete or inappropriate treatment, and contributes to the emergence of drug resistance. Ensuring standardized case management across all levels of care is essential to mitigate these risks and improve treatment outcomes [5]. Deviations from these standards can lead to suboptimal patient outcomes, increased drug resistance, and a greater burden on the healthcare system. Yet in remote areas with limited access to reliable microscopy or rapid diagnostic tests (RDTs), it is challenging empirical treatment and potential misdiagnosis [2]. In a retrospective analysis of 3203 blood smears, the rate of false-positive malaria diagnosis was a staggering 75.6%, and a 0.01% false-negative rate. This study, which re-evaluated smears from 95 peripheral health facilities and included prospective validation with rapid diagnostic tests, exposed poor skills among general practitioners (GPs) and general microscopists (GMs), with 43% of GPs and 44% of GMs failing to make a single true-positive malaria diagnosis [6].

Sudan’s National Malaria Control Program (NMCP), supported by international partners, has updated its malaria guidelines (2023) to align with global best practices. The revised guidelines emphasize parasitological confirmation (microscopy or RDTs) for all suspected cases, eliminating clinical diagnosis. For treatment, parenteral artesunate is now the first-line therapy for severe malaria, replacing quinine, which is now reserved as second-line. In uncomplicated cases, artemether-lumefantrine (AL) remains first-line, with dihydroartemisinin-piperaquine (DHAP) as second-line. Other changes were also made to the management of malaria in pregnant women [7].

To evaluate the real-world implementation of these guidelines, an audit was conducted in Dongola Specialized Hospital with the primary objective of assessing the current level of adherence to established national guidelines for malaria diagnosis and treatment among healthcare professionals. By evaluating key parameters, this study aimed to identify strength areas, specific gaps in practice, and measure the impact of targeted interventions designed to improve the quality of malaria management.

## Materials and methods

Study design and setting

This clinical audit was conducted in the Department of Internal Medicine at Dongola Teaching Hospital, Sudan, between May 27 and June 28, 2025. The primary objective was to assess physicians’ adherence to the 2023 Sudan Malaria Case Management Protocol and evaluate the impact of targeted educational interventions on guideline compliance.

Audit Criteria and Definitions

The audit examined three overarching domains: diagnostic accuracy, treatment appropriateness, and protocol compliance across malaria case management standards.

Diagnostic accuracy was defined as the alignment of malaria diagnosis with national guidelines, emphasizing parasitological confirmation using microscopy or RDTs and the correct identification of Plasmodium species.

Treatment appropriateness assessed whether prescribed therapies conformed to recommended first-line and second-line agents based on malaria severity, species type, and patient subgroups (e.g., pregnant women, severe malaria cases).

Protocol compliance referrs to the extent to which physicians followed updated national standards, including correct artesunate administration routes, timing of treatment initiation, and proper use of intermittent preventive treatment in pregnancy (IPTp).

Audit Population and Sample Size

The audit involved physicians actively managing malaria cases, including medical interns, general practitioners, residents, and consultants. A total of 81 participants were included across two audit cycles: 42 physicians were assessed in the first cycle (baseline), and 39 were reassessed in the second cycle post-intervention. A full-coverage approach was used to include all eligible physicians during the study period. Participants were unaware of the audit to mitigate observer bias and better reflect routine clinical practice.

Data Collection and Analysis

Data were collected using a pre-tested, validated questionnaire (Appendix) designed to evaluate adherence across 14 malaria management standards outlined in the 2023 national protocol [8]. The questionnaire captured responses on diagnostic practices, treatment regimens, and special population management. To ensure consistency, the tool was administered uniformly across both audit cycles.

Analysis was conducted using Microsoft Excel (Microsoft Corporation, Redmond, WA, USA). Compliance rates were calculated for each parameter and compared between the two cycles to assess the effectiveness of the implemented interventions.

Audit cycles

Pre-intervention Phase (First Cycle)

The first cycle involved a retrospective assessment of physicians' knowledge and application of malaria management guidelines. Data from this phase established the baseline adherence rates and highlighted key deficiencies in clinical practice.

Intervention Phase

To address identified gaps, a structured intervention strategy was implemented. This included both online and in-person educational sessions, each lasting approximately three hours and delivered in a modular format. The training focused on key areas such as diagnostic standards, treatment algorithms, and management of special populations. Core learning objectives emphasized improving documentation accuracy, reinforcing correct artesunate administration, and ensuring proper use of IPTp. Instructional materials comprised slide decks, annotated clinical scenarios, and compliance checklists aligned with the 2023 Sudan Malaria Case Management Protocol. To reinforce learning, educational posters summarizing key management steps were placed across wards and staff areas. Additionally, printed copies of the updated national guidelines were distributed to all departments to enhance accessibility and encourage consistent reference during clinical practice.

Post-intervention Phase (Second Cycle)

Following the intervention period, the second cycle commenced using the same questionnaire. This phase aimed to evaluate improvements in physicians’ adherence to national standards and measure the impact of training and reinforcement tools.

Ethical considerations

Ethical approval for this clinical audit was obtained through the Dongola Specialized Hospital Ethics Committee under IRB reference number 2025/Au/036. All responses were anonymized prior to analysis to uphold confidentiality standards.

## Results

First cycle results (N = 42)

The initial assessment, conducted with 42 doctors, revealed varying levels of adherence to malaria treatment standards. Protocol adherence was 13 (30.95%), while identification of the most common Plasmodium species was 31 (73.81%). Treatment timeliness was recorded at 19 (45.24%), and the use of gold standard diagnostics was at 24 (57.14%). Adherence to first-line treatment with AL for P. falciparum was notably high at 38 (90.48%). For P. vivax, primaquine acknowledgment was at 27 (64.29%). First-line treatment for severe malaria, considering no contraindications, was adhered to in 33 (78.57%) of cases. Uncomplicated malaria in pregnancy (MIP) during any trimester saw 25 (59.52%) adherence to first-line treatment. The recommended site for intramuscular (IM) injection of artesunate was correctly identified in only 13 (30.95%) of instances, and second-line treatment for uncomplicated MIP during the second and third trimesters had the lowest adherence at 10 (23.81%). Finally, IPTp correct drug sulfadoxine/pyrimethamine (SP) was chosen correctly in 18 (42.86%) of cases, and congenital malaria treatment with IV artesunate was at 25 (59.52%) as presented in Table 1.

**Table 1:** Comparison between the first and second cycles, showing the respective percentage of improvement. AL: artemether-lumefantrine, IPTp: intermittent preventive treatment in pregnancy, SP: sulfadoxine/pyrimethamine

Standards	First cycle, N = 42	Second cycle, N = 39	Percentage of improvement
Protocol Adherence	13(30.95%)	35(89.74%)	58.79%
The most common Plasmodium species	31(73.81%)	36(92.31%)	18.50%
Treatment Timeliness	19(45.24%)	33(84.62%)	39.38%
Gold Standard Diagnostic	24(57.14%)	37(94.87%)	37.73%
Essential for a microscopy report of Giemsa-stained blood films	32(94.87%)	36(92.31%)	16.12%
First-Line Treatment (AL for P. falciparum)	38(90.48%)	39(100.00%)	9.52%
Primaquine for P. vivax	27(64.29%)	35(89.74%)	25.45%
First line for severe malaria considering no contraindications	33(78.57%)	33(84.62%)	6.05%
First line treatment for uncomplicated malaria in pregnancy (MIP) during any trimester	25(59.52%)	30(76.92%)	17.40%
The recommended site for intramuscular (IM) injection of artesunate	13(30.95%)	31(79.49%)	48.54%
The second-line treatment for uncomplicated MIP during the 2nd and 3rd trimesters	10(23.81%)	25(64.10%)	40.29%
IPTp Correct Drug (SP)	18(42.86%)	36(92.31%)	49.45%
Congenital Malaria (IV Artesunate)	25(59.52%)	32(82.05%)	24.69%
Most serious complication of quinine	35(83.33%)	36(92.31%)	8.98%

Second cycle results (N = 39)

Following the implementation of interventions, a second assessment involving 39 doctors demonstrated improvements across most standards. Protocol adherence surged to 35 (89.74%), and the identification of the most common Plasmodium species improved to 36 (92.31%). Treatment timeliness significantly increased to 33 (84.62%), and the use of gold standard diagnostics reached 37 (94.87%). Adherence to first-line treatment with AL for P. falciparum further improved to 100%, indicating good compliance. Primaquine for P. vivax rose to 35 (89.74%). First-line treatment for severe malaria, considering no contraindications, increased to 33 (84.62%). For uncomplicated MIP during any trimester, first-line treatment adherence improved to 30 (76.92%). The correct site for IM injection of artesunate showed a substantial rise to 31 (79.49%), and second-line treatment for uncomplicated MIP during the second and third trimesters increased to 25 (64.10%). IPTp correct drug (SP) recognition improved to 36 (92.31%), and congenital malaria treatment with IV artesunate reached 32 (82.05%) as shown in Table 1.

## Discussion

The comparative analysis between the first and second cycles indicates an enhancement in adherence to malaria treatment guidelines. The results also revealed that while doctors had good knowledge of malaria management, they were not adequately aware of many changes in the national guideline. The second cycle results proved that educational programs can help fill the theory-practice gap. This is particularly essential in a dynamic field like malaria management. Updates to the guideline reflect drug resistance patterns, new diagnostics, and improved treatments. The initial lack of knowledge regarding guideline changes, despite a good foundational understanding, highlights a common challenge in healthcare settings. This audit showed significant gaps not only in awareness of updated guidelines but also in their consistent application in clinical practice. The absence of systematic assessment in adherence to national malaria guidelines further complicates efforts to address deficiencies.

Regional audits frequently highlight challenges in adherence to malaria guidelines, particularly concerning specific populations. In Uganda, an audit revealed that children under 18 had a lower rate of inappropriate management (10.7%) compared to older adults (18-30 years: 20%; over 30 years: 21.4%) [9]. Also, an audit from Malawi showed inconsistencies in adherence to treatment guidelines in children, where 102 (29.2%) patients did not receive the appropriate treatment [10]. Similarly, an audit conducted in Nigeria investigated pediatric malaria guidelines across private and public healthcare centers and found that 58% of cases in private facilities and 47% in public centers received the correct oral antimalarial doses [11]. Similar to these findings, our study's initial cycle revealed low adherence to guidelines for specific populations (Table 1). Notably, adherence to congenital malaria treatment was 25 (59.52%), second-line treatment for uncomplicated malaria in pregnancy was 10 (23.81%), and IPTp drug choice was 18 (42.86%).

Beyond population-specific management, adherence to diagnostic and general treatment guidelines showed some disparities. The Ugandan audit, for example, reported that 50 patients (84.7%) were appropriately diagnosed prior to treatment, and the remaining patients were managed without a prior diagnosis or contrary to established guidelines [9]. In Ethiopia, another audit showed that while strong adherence (82.9%) to ideal Plasmodium vivax treatment was observed, significant diagnostic gaps persisted. Only 19.4% of malaria-suspected patients were investigated according to recommended diagnostic protocols [12]. In our first cycle, 27 (64.29%) of participants chose the correct management for P. vivax, and the use of gold standard diagnostics was 24 (57.14%). Regarding general treatment, the initial adherence to first-line AL for P. falciparum was already high at 38 (90.48%) and reached 39 (100%) in the second cycle (Table 1). Similarly, a multi-center study reported 64.8% appropriate ACT dosing, with 23.5% incorrect doses and 8.9% completely inappropriate treatments [13].

Unlike the single-cycle audits mentioned above, our study incorporated baseline post-intervention evaluation. This allowed us to measure the impact of educational efforts on adherence. Moreover, our audit covered a broader range of malaria management parameters, including specific diagnostic aspects, various treatment regimens (first-line, severe, P. vivax), and critical population-specific management (MIP, congenital malaria, IPTp). This provides a more holistic view of adherence across the entire patient pathway compared to studies focusing predominantly on diagnosis.

To enhance clinical competency in critical areas such as malaria management for specific populations, intramuscular artesunate administration and alternative treatment protocols, educational programs should emphasize training sessions that incorporate more case-based learning, simulations, and supervised clinical practice opportunities. Improving diagnostic accuracy remains paramount in Sudan's clinical settings, where false-positive malaria results remain prevalent. Our results suggest that combining practical microscopy training with robust quality control mechanisms could significantly reduce diagnostic errors. Furthermore, embedding routine clinical audits into existing hospital quality frameworks appears essential for maintaining these improvements over time. Future audits should assess guideline adherence across Sudanese institutions and regions. A comprehensive multi-center analysis could reveal challenges across regions, thereby improving malaria management countrywide. These audits, leveraging robust quality improvement methodologies, should actively explore underlying barriers to adherence, moving beyond simple compliance monitoring to understand the root causes of practice gaps. Such assessments should also incorporate structured feedback mechanisms that encourage reflective practice and support clinicians in adopting treatment guidelines.

While this study provides insight into doctors' adherence to the updated malaria protocol, it exclusively relies on self-reported data, rather than objective measures of clinical practice. This method risks bias, as doctors might overreport their adherence. Furthermore, the intervention primarily focused on didactic teaching (online and on-site sessions, educational posters) and did not include practical training, which may limit its effectiveness in translating knowledge into actual clinical skills or behavioral change. Moreover, while the study's duration was long enough to demonstrate an initial change in knowledge, it might have been too short to evaluate the retention of that knowledge over time.

Strengths and limitations

This is the first audit to systematically assess physicians’ adherence to the updated 2023 Sudan Malaria Case Management Protocol. The audit provides a foundational reference for future multi-center evaluations and policy implementation tracking. However, this audit was conducted in a single hospital, which may restrict the applicability of its findings to broader healthcare settings across Sudan. Variations in institutional resources, physician training, and patient demographics could influence adherence patterns not captured in this study. Additionally, the lack of long-term follow-up limits the ability to assess sustained compliance with the 2023 Sudan Malaria Case Management Protocol or the impact of any subsequent interventions.

## Conclusions

This two-cycle audit demonstrated improvements in adherence to national malaria diagnosis and treatment guidelines among healthcare professionals in Sudan. The targeted interventions implemented after the first cycle proved highly effective in addressing identified gaps, leading to increases in compliance across almost all assessed standards. These positive shifts underscore the critical role of continuous education in enhancing the quality of malaria management. Sustained efforts are essential to maintain these improvements and further strengthen the delivery of high-quality malaria care.

## Appendices

Assessing Physicians’ Adherence to the 2023 Sudan Malaria Case Management Protocol

1. Current position

House Officer

Medical Officer

Registrar

Consultant

Other:

2. Year of Graduation

3. Department

General Surgery

Obstetrics & Gynaecology

General Medicine

Paediatrics

Other:

4. Email

5. Which version of the national malaria case management protocol in Sudan have you most recently read and currently use as a reference in your clinical practice?

2016

2017

2022

2023

I am not sure

6. Please indicate which Plasmodium species is most commonly responsible for malaria infections in Sudan:

Plasmodium vivax

Plasmodium falciparum

Mixed infections (P. falciparum and P. vivax)

Plasmodium malariae

7. What is the recommended timeframe for initiating effective treatment for uncomplicated Plasmodium falciparum malaria to prevent progression to severe disease?

Within 6-12 hours of symptom onset

Within 24-48 hours of symptom onset

Within 3-5 days of symptom onset

Only after confirmation of severe symptoms

8. Which of the following is considered the gold standard method for parasitological diagnosis of malaria?

Rapid Diagnostic Tests (RDTs)

Quality-assured light microscopy

Clinical symptoms alone

Serological antibody tests

9. Which of the following features are considered essential for a microscopy report of Giemsa-stained blood films to be complete and accurate?

Presence of malaria parasite (seen or not seen)

Parasite species

Stages of the parasite

Parasite count by microliter (μL)

All of the above

10. What is the first-line treatment for uncomplicated Plasmodium falciparum malaria in Sudan?

Chloroquine tablets

Artemether-lumefantrine (AL) tablets

Dihydroartemisinin + piperaquine (DHAP) tablets

Quinine tablets

11. Which drug should be administered after artemether-lumefantrine (AL) to achieve radical cure in uncomplicated Plasmodium vivax malaria?

Chloroquine

Primaquine

Quinine

DHAP

12. Which of the following are considered clinical signs of complicated (severe) malaria?

Glasgow Coma Score < 11 in adults

More than two convulsions episodes within 24 hours

Jaundice

Pulmonary edema

Mild headache

Respiratory distress (acidotic breathing)

Shock

Fever below 37°C

Prostration (severe generalized weakness)

Continuous vomiting

Mild fatigue

Hypotension

13. Which of the following are considered laboratory findings indicative of complicated (severe) malaria?

Severe anemia (Hb ≤ 5 g/dL in children; Hb < 7 g/dL in adults)

Hypoglycemia (< 2.2 mmol/L or < 40 mg/dL)

Plasma bicarbonate > 15 mmol/L

Hyperlactatemia (lactate ≥ 5 mmol/L)

Hyperparasitemia (P. falciparum > 10%)

Renal impairment (Serum creatinine > 265 μmol/L or blood urea > 20 mmol/L)

Abnormal bleeding

Mild anemia (Hb > 10 g/dL)

Hypernatremia

Hypokalemia

Low lactate levels

14. Considering no contraindication, what is the treatment of choice (1st line) for severe malaria?

Oral artemether-lumefantrine (AL)

Intravenous artesunate

Intravenous quinine

Intramuscular quinine

15. What is the first-line treatment for uncomplicated malaria in pregnancy (MIP) during any trimester?

Quinine tablets

Artemether-lumefantrine (AL) tablets

Dihydroartemisinin-piperaquine (DHAP) tablets

Sulfadoxine-pyrimethamine (SP)

16. What is the recommended site for intramuscular (IM) injection of artesunate?

Deltoid muscle

Anterior thigh

Gluteal muscle

17. What is the second-line treatment for uncomplicated MIP during the 2nd and 3rd trimesters?

Artemether-lumefantrine (AL) tablets

Dihydroartemisinin-piperaquine (DHAP) tablets

Quinine tablets

Sulfadoxine-pyrimethamine (SP)

18. What is the drug of choice for Intermittent Preventive Treatment of Malaria in Pregnancy (IPTp)?

Artemether-lumefantrine (AL) tablets

Dihydroartemisinin-piperaquine (DHAP) tablets

Quinine tablets

Sulfadoxine-pyrimethamine (SP)

19. What is the treatment of choice for congenital malaria?

Intravenous artesunate

Intravenous quinine

Intramuscular quinine

Intramuscular artesunate

20. Which of the following is the most serious and frequent adverse effect associated with quinine treatment for malaria?

Vomiting

Nausea

Hypokalemia

Hypoglycemia
